# Supplementary material for: Balanced Crystalloids versus Normal Saline in Adults with Sepsis: A Comprehensive Systematic Review and Meta-Analysis
Source: J Clin Med. 2022 Apr 1;11(7):1971. doi: 10.3390/jcm11071971 (PMC8999853; doi:10.3390/jcm11071971)
Supplement: Supplementary file 1 [file jcm-11-01971-s001.zip › jcm-1639872-supplementary/Supplementary material.pdf]

Supplementary Appendix

Balanced Crystalloids versus Normal Saline in Adults with Sepsis: A Comprehensive Systematic Review and Meta-Analysis

Supplementary tables

Title	Page
Supplementary Table S1: Search strategy used in each database searched.	2
Supplementary Table S2: Quality assessment of the included studies in the meta-analysis.	3-4

Supplementary figures:

Title	Page
Supplementary Figure S1: Leave-one-out sensitivity analysis for overall mortality, acute kidney injury, and need for renal replacement therapy.	5
Supplementary Figure S2: Leave-one-out sensitivity analysis for overall mortality by exclusion of the study by Shaw et al.	6
Supplementary Figure S3: Funnel plot showing publication bias analysis of studies that compared balanced crystalloids versus normal saline regarding overall mortality.	7

Supplementary Table S1: Search strategy used in each database searched.

Database	Search Strategy	Articles retrieved
PubMed/MEDLINE	("sepsis"[MeSH Terms] OR "sepsis"[All Fields] OR ("shock, septic"[MeSH Terms] OR ("shock"[All Fields] AND "septic"[All Fields]) OR "septic shock"[All Fields] OR ("septic"[All Fields] AND "shock"[All Fields]))) AND ("saline solution"[MeSH Terms] OR ("saline"[All Fields] AND "solution"[All Fields]) OR "saline solution"[All Fields] OR ("normal"[All Fields] AND "saline"[All Fields]) OR "normal saline"[All Fields] OR (("isotonic"[All Fields] OR "isotonically"[All Fields] OR "isotonicity"[All Fields] OR "isotonics"[All Fields]) AND ("saline solution"[MeSH Terms] OR ("saline"[All Fields] AND "solution"[All Fields]) OR "saline solution"[All Fields] OR "saline"[All Fields] OR "salines"[All Fields]))) AND ((("balance"[All Fields] OR "balanced"[All Fields] OR "balances"[All Fields] OR "balancing"[All Fields]) AND ("crystalloid solutions"[MeSH Terms] OR ("crystalloid"[All Fields] AND "solutions"[All Fields]) OR "crystalloid solutions"[All Fields] OR "crystalloid"[All Fields] OR "crystalloids"[All Fields] OR "crystalloidal"[All Fields])) OR ("ringer s lactate"[MeSH Terms] OR ("ringer s"[All Fields] AND "lactate"[All Fields]) OR "ringer s lactate"[All Fields] OR ("lactated"[All Fields] AND "ringer"[All Fields]) OR "lactated ringer"[All Fields]) OR "plasmalyte"[All Fields])	97
Embase	('sepsis'/exp OR sepsis OR 'septic shock'/exp OR 'septic shock' OR (septic AND ('shock'/exp OR shock))) AND ('normal saline'/exp OR 'normal saline' OR (normal AND ('saline'/exp OR saline)) OR 'isotonic saline' OR (isotonic AND ('saline'/exp OR saline))) AND ('balanced crystalloids' OR (balanced AND crystalloids) OR 'lactated ringer' OR (lactated AND ringer) OR 'plasmalyte'/exp OR plasmalyte)	156
Web of Science	(sepsis OR (septic shock)) AND ((normal saline) OR (isotonic saline)) AND ((balanced crystalloids) OR (lactated ringer) OR plasmacyte)	102

Supplementary Table S2: Quality assessment of the included studies in the meta-analysis.

Cohort studies	Selection				Comparability	Outcome			Quality score
	Representativeness of the exposed cohort	Selection of non-exposed cohort	Ascertainment of exposure	Demonstration that outcome of interest was not present at start of study	Comparability of the cohorts on the basis of design or analysis	Assessment of outcome	Was follow up long enough for outcomes to occur	Adequacy of follow up cohorts	
Duffy, 2018	1	1	1	1	1	1	0	1	7
Jaynes, 2017	1	1	1	1	1	1	0	1	7
Limapichat, 2021	1	1	1	1	0	1	0	1	6
Mao, 2018	1	1	1	1	1	1	0	0	6
Raghunathan, 2014	1	1	1	1	1	1	0	1	7
Shaw, 2017	1	1	1	1	1	1	0	0	6
Tseng, 2021	1	1	1	1	1	1	1	0	7

Randomized controlled studies	Randomization		Blinding		Withdrawals	Quality score
	Was the study described as randomized?	Was the randomization appropriate?	Was the study described as double blind?	Was the double blinding appropriate?	Was there a description of withdrawals and dropouts?	
Annane, 2013	1	1	0	0	1	3
Finfer, 2022	1	1	1	1	1	5
Golla, 2020	1	1	0	0	1	3
Pagano, 2020	1	1	0	0	1	3
Semler, 2017	1	1	0	0	1	3
Semler, 2018	1	1	0	0	1	3

Young, 2015	1	1	1	1	1	5
Zampieri, 2021	1	1	1	1	1	5

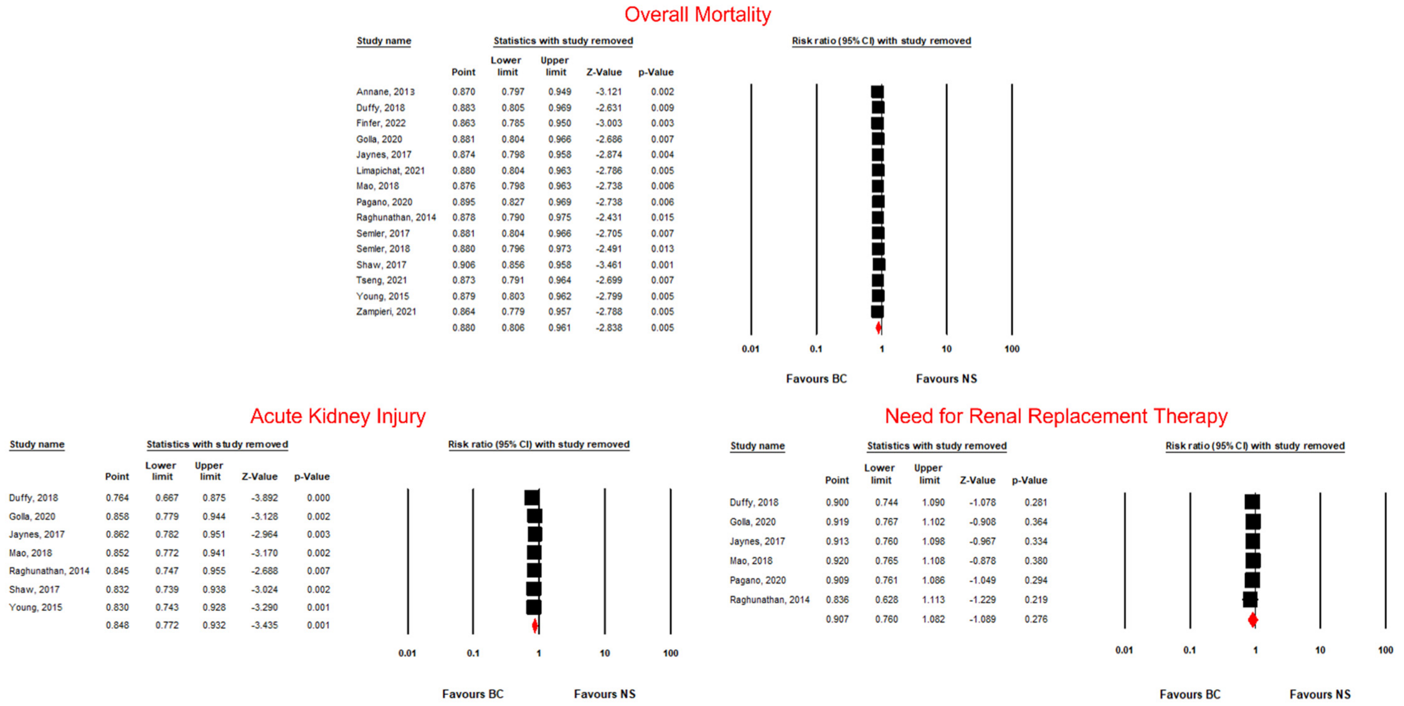

Supplementary Figure S1: Leave-one-out sensitivity analysis for overall mortality, acute kidney injury, and need for renal replacement therapy.

Study or Subgroup	BC Events	BC Total	NS Events	NS Total	Weight	Risk Ratio M-H, Random, 95% CI
Annane, 2013	16	37	197	557	2.1%	1.22 [0.83, 1.80]
Duffy, 2018	68	680	66	538	3.0%	0.82 [0.59, 1.12]
Finfer, 2022	276	1068	265	1026	12.4%	1.00 [0.87, 1.16]
Golla, 2020	29	80	35	80	2.1%	0.83 [0.57, 1.21]
Jaynes, 2017	37	201	38	209	1.9%	1.01 [0.67, 1.52]
Limapichat, 2021	2	20	14	100	0.2%	0.71 [0.18, 2.90]
Mao, 2016	48	98	54	100	4.1%	0.91 [0.69, 1.19]
Pagano, 2020	8	35	28	49	0.7%	0.40 [0.21, 0.77]
Raghunathan, 2014	659	3365	768	3365	24.0%	0.86 [0.78, 0.94]
Semler, 2017	27	130	33	130	1.6%	0.82 [0.52, 1.28]
Semler, 2018	294	1167	344	1169	14.3%	0.86 [0.75, 0.98]
Tseng, 2021	114	302	263	636	9.3%	0.91 [0.77, 1.08]
Young, 2015	6	41	7	43	0.3%	0.90 [0.33, 2.45]
Zampieri, 2021	453	970	498	1017	24.1%	0.95 [0.87, 1.05]
**Total (95% CI)**		**8194**		**9019**	**100.0%**	**0.91 [0.86, 0.96]**
Total events	2037		2610			

Heterogeneity: Tau² = 0.00; Chi² = 14.66, df = 13 (P = 0.33); I² = 11%

Test for overall effect: Z = 3.43 (P = 0.0006)

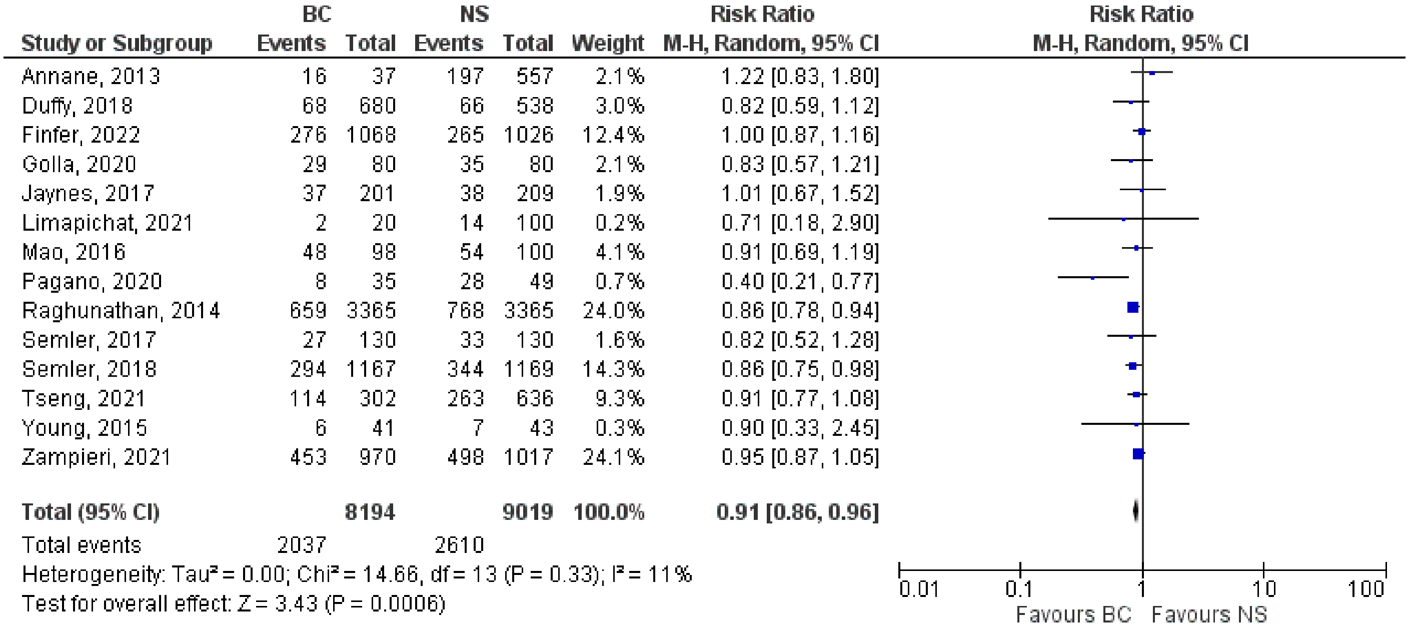

Supplementary Figure S2: Leave-one-out sensitivity analysis for overall mortality by exclusion of the study by Shaw et al.

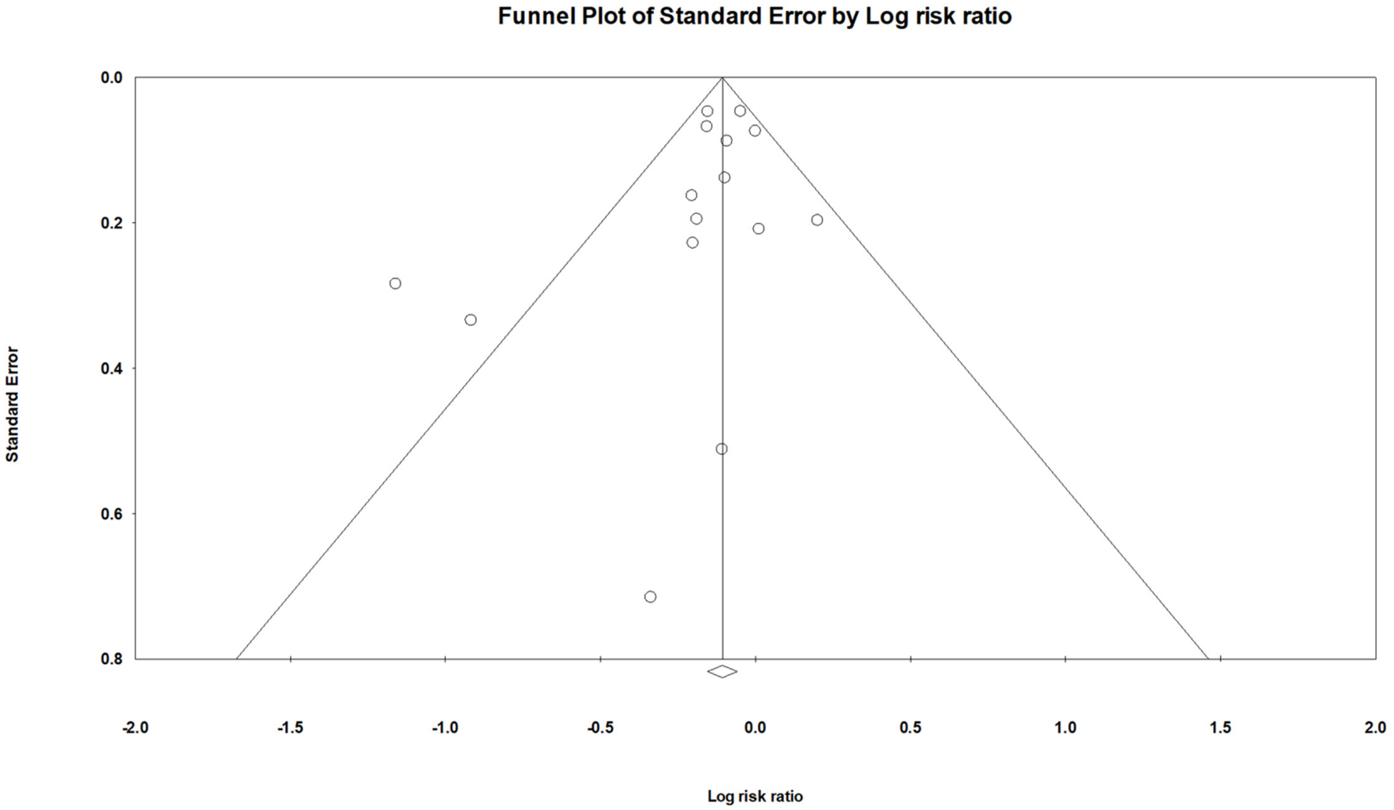

Egger's regression intercept

Intercept	-0.80915
Standard error	0.58636
95% lower limit (2-tailed)	-2.07590
95% upper limit (2-tailed)	0.45761
t-value	1.37994
df	13.00000
P-value (1-tailed)	0.09544
P-value (2-tailed)	0.19088

Supplementary Figure S3: Funnel plot showing publication bias analysis of studies that compared balanced crystalloids versus normal saline regarding overall mortality.
